# Editorial: Neurobiological foundations of cognition and progress toward artificial general intelligence

**DOI:** 10.3389/fnsys.2026.1967487

**Published:** 2026-09-14

**Authors:** Yan Yufik, Robert Kozma, James Kroger

**Affiliations:** 1 Virtual Structures Research Inc., Potomac, MD, United States; 2 The University of Memphis, Memphis, TN, United States; 3 Department of Biological Sciences, The University of Memphis, Memphis, TN, United States; 4 Department of Interdisciplinary Studies, New Mexico State University, Las Cruces, NM, United States

**Keywords:** energy expenditures, intelligence, neuronal packet, neuronal resource maneuvering, self-organization, understanding, meaning creation

This Research Topic concludes a trilogy in Frontiers Systems Neuroscience, with the preceding two issues addressing Self-Organization in the Nervous System (2016) and Understanding Capacity in the Human and the Machine (2021). In this editorial, we review contributions in volume 1 along with some other findings during the 2016–2026 period and apply the results to re-iterate the problems that motivated the trilogy and to invite further discussions in volume II.

The problems are as follows: (a) what is intelligence, (b) how human intelligence differs from that in the other species, (c) how could intelligence emerge in the physical universe, (d) what is the future of AI?, and (e) The last decade has witnessed breakthrough advances in machine learning methods rooted in the ideas of digital neurons and perceptron dating back to the mid 20th century, what factors have been responsible for these advances? To facilitate discussion, we outline opinions and hypothetical solutions that are intended as discussion starters and encompass theoretical foundations, synopsis of contributions in this volume and a new synthesis.

First, the opinions. (a) According to a definition attributed to Jean Piaget “intelligence is what you use when you don't know what to do, when neither innateness nor learning has prepared you for the particular situation” (Calvin, 1996; p. 1) Novel situations engage the understanding capacity in a dual function of constructing adequate responses and overcoming the inertia of prior learning (the legacy mind set) interfering with the construction. (b) All species are capable of learning while the understanding capacity is uniquely human. (c) Intelligence mechanisms are hypothesized to be integral to those underlying the emergence of life and involving self-organization in networks of interacting elements where parts of networks fold into stable units separated by an active surface (“membrane”) from their surrounds (Friston, 2010). (d) The future of AI involves transition from machine learning to machine understanding centered on the approximation of meaning creation in self-organizing neuronal networks. (e) Machine learning methods are computationally demanding. Their successes have been due to a confluence of two developments: ingenious heuristics to minimize computational demands and a trillion-fold increase of hardware efficiency. Machine understanding will employ moderate capacity platforms with low energy demands to deliver functions inaccessible to learning (Yufik and Yufik, 2026). Next, the hypotheses.

## 1 The foundations

In the history of psychology, behaviorism was reducing intelligence to conditioning while cognitivism has been equating intelligence to computation. The “Bayesian intelligence” school of thought conceptualizes brain as a “prediction machine.” Theories of understanding conceptualize human brain as a biophysical system for the production of meaning and pivot on the ideas of “neuronal packets” and “wave packets” (Yufik and Yufik, 2026; Yufik, 1998a,b; Freeman, 2000; Kozma and Freeman, 2026). The former idea coheres with the key insight in Gestalt psychology:

“Sensory units have acquired names, have become symbolic and known to have practical uses while they have existed as units before any of these further facts were added. … it is precisely the original segregation of circumscribed wholes which makes it possible for the sensory world to appear so utterly imbued with meaning” (Kohler, 1947; p. 82).

The “wave packets” idea attributes production of meaning to amplitude- and phase- modulated waves of activity percolating through systems of interacting neuronal populations. Suggestions for the integration of the two conceptions are stated in Section 3.

Understanding involves “apprehending general relations in multitudes of particulars” (Webster's Dictionary). A parable of blind men and an elephant comes in handy: meaning is produced in constructing relations that integrate disparate reports into a coherent whole (assume a blind analyst charged with the task). Integration enables explanation and prediction: elephants behave differently from any of the entities in the reports. Piaget's theory characterizes understanding as:

“The system of operational co-ordinations that transform objects of thought in the same way as actions transform material objects… in order to understand, one must be able to construct in thought indefinite series … and treat the series one has actually observed as just one sector of an unlimited range of possibilities” (Piaget, 1975, 1978, p. 222).

Another critical insight in psychology concerns the dynamics of (the “movement of consciousness” in) coordinating some object Z with other objects, starting will accessing Z:

“Through hovering of the attention in the neighborhood of the desired object, the accumulation of associates becomes so great that the combined tension of their neural processes break through the bar, and the nervous wave pours into the tract which has so long been awaiting its advent… The activities in Z will at first be a mere tension … until combined irradiations upon Z succeed in helping the tension there to overcome the resistance and is arousing Z to full activity” (James, 1890, v.1, p. 586).

Finally, the most fundamental, cornerstone idea in psychology and neuroscience is the idea of neuronal assemblies by Donald Hebb, conceptualizing groups of neurons as the basic functional units in the neuronal system. A critical insight concerns interaction between assembles in the formation of percepts (e.g., assemblies a, b, and c interact in forming the percept of triangle ABC):

“…simultaneous activity in a, b, c would establish facilitation between them, through their chance anatomical interconnections and the enlargement of synaptic knobs… The resulting superordinate system must be essentially a new one, by no means a sum of hooking together of a, b, and c… a better notation for the new structure is t: the assembly of cells whose activity… is perception of the triangle as a distinctive whole” (Hebb, 1947; p. 97)

In the emerging integrated picture so far, bringing together in imagination two “segregated wholes” appears to be one of the basic operations in meaning creation. Unsurprisingly, a received theory of language understanding places operation Merge (combining some objects Z and some X into a unit) at the core of the capacity (Chomsky, 2014; Berwick and Chomsky, 2016). We will return to these notions in Section 3, after re-centering the discussion as follows.

Production of meaning is coextensive with making choices (which Z, which X?): automatisms admit no choices, choices are drilled in from the outside in learning and are decided internally in understanding. An array of 10 objects affords 1,024 choices, accounting for permutations (A after B is not the same as B after A) increases that number to 3, 628, 800. Boundary barriers (or “bars” James, 1890, v.1, p. 586) turn culling through combinations into steeplechase running, multiple objects (e.g., 32 chess pieces) yield astronomically large combinatorial spaces: how can the human system run steeplechase marathons and real time sprints in such spaces while subsisting on miniscule energy budgets? “Operational coordination” (Piaget, 1978, p. 222) transforms combinations into coherent structures (models) amenable to coordination with other models: what mechanisms drive the expanding coherence? Section 3 ventures a hypothesis: understanding compresses combinatorial spaces into minute volumes imbued with meaning. Section 2 highlights connections between the articles in this volume, the questions above and the theory in Section 3.

## 2 Contributions

Davis et al. analyze EEG data to assess the degree of coherence in neuronal processes, on the assumption that high coherence corresponds to achieving mental clarity and entering into a meditative state of mind. Following the Global Workspace Theory, the paper attributes achieving coherence to global information broadcasting and active integration, with the spotlight of consciousness illuminating the salient contents. Conscious awareness can also include “silent” elements having no reportable sensory content. Global coherence is equated to synchronization entailing reduction of entropy.

Dodig-Crnkovic presents a broadly conceptualized analysis of biological cognition and AI, addressing the possible origins and observable manifestation of intelligence in the physical universe. Within this all-encompassing framework (Info-Computational Framework, or ICON) the mind is viewed as a material phenomenon in a continuum stretching from inanimate substances to primitive organisms and up to sentient beings. The article includes definitions of general notions, for example, “information is a difference that makes a difference for the regulation of system environment interactions,” etc. The intended meaning of such definitions is explicated in the analysis, including validation approaches.

Joseph et al. offer an elegant interpretation of embryogenesis dynamics in terms of predictive error minimization. The predictive error minimization framework is given a dual application: an explanatory principle in a theory of self-organization dynamics in the embryo and a mathematical tool for predicting that dynamics from data (embryo viability, etc.) The proposed theory views synaptically linked neurons as coupled oscillators, self-organization drives synaptic fluxes toward equilibrium in a synchronized state where each couple functions as an element in Markov blanket that maintains equilibrium in the system and causes convergence toward error minimization.

Farkaš et al. argue that Large Language Models (LLN) fail to address the symbol grounding problem and operate strictly on the basis of statistical regularities. They contend that closing conceptual gaps between symbolic tokens, neural representations, and embodied experiences will require a theoretical framework accounting for the integration of continuous sensory flows, goal-directed behavior and neural learning.

Grossberg reviews developments in Adaptive Resonance Theory (ART) since the time of its inception and presents new results, comparing performance in ART to that in LLM. The article argues that progress toward AGI will lead to Autonomous Adaptive Intelligence (AAI) systems implementing the core ART intuition: learning proceeds in an interplay between bottom-up and top-down processes. Comparing AAI to LLM highlights the limitations of the latter rooted in the limitations of deep learning.

Kirkpatrick argues insightfully that human intelligence is fundamentally grounded in motion and asserts that AGI will thoroughly integrate motion and cognition and thus escape from the circular logic inherent in the computational metaphor. In a tour de force analysis, the paper traces successes and shortcoming of the metaphor to McCullogh-Pitts models of neurons inspired by the metaphor. A critical limitation of such models is manifested in their ability to emulate proofs of mathematical propositions but not the processes that underlie the understanding of those proofs. The paper makes constructive suggestions for the development of biologically inspired AGI.

Rachman and Pashea associate human intelligence with problem solving in diverse contexts under a general objective of minimizing uncertainty. The authors observe that the uncertainty minimization criteria apply to all species while the use of symbolic systems allows humans to expand the contexts beyond the biologically fixed constraints. Framing the challenge facing AGI as creating systems that are aligned with human values and match human cognitive flexibility, the article reverts Active Inference as a promising venue toward meeting that challenge. A novel idea in their approach is the placement of moral values at the apex of predictive hierarchies. A formalism is proposed (“transcendental inference”) that decomposes variational free energy into accuracy and complexity terms and expresses conditions for social agreement as maximizing accuracy (benefits) while minimizing complexity (costs).

## 3 The synthesis

A theory of understanding requires switching from the “intelligence through learning” mindset to the “intelligence through understanding” mindset: the former one is expressed in the idea of perceptron (a fixed neuronal structure adapting through feedback to repetitive stimuli bundles) while the latter one envisions a self-organizing neuronal pool adapting to varying stimuli streams where stimuli compositions might be never twice the same. We will use the Virtual Associative Structures (VaST) proposal (Yufik, 1998a) to illustrate the idea, starting with the key assumptions.

- The pool—environment interactions seek to secure steady energy inflows from the energy-carrying stimuli. The evolution of intelligence involves minimization of expenditures (caloric costs and dissipative losses) in securing supplies at life-sustaining levels. In the human, the process is self-adaptive (Yufik, 1998b) in that it can decouple itself from the inputs and invest energy into re-organization facilitating further expenditure reductions.
- Expenditure minimization engages three primary mechanisms: neuronal packets, relational neurons and complexes of coordinated packets (models). Neuronal packets self-organize in associative networks when tightly associated subnets fold into functional units separated by energy barriers from the surrounding net. Models form in hierarchies of networks when upper level complex neurons facilitate coordination between low level packets while the growing coordination facilitates activation in the corresponding upper level neurons.
- Packets and models are self-organizing units, matching stimuli streams by units and models produces collapses in the combinatorial space reducing intractable multitudes of possible combinations to a handful of meaningful candidates. Crucially, spurious alternatives outside of such handfuls are not rejected but remain beyond the boundaries of awareness.
- Perceptron learns automatic responses to contiguous stimuli bundles; deep learning compresses multiple such automatisms into one perceptron. Neuronal packets are “molecules of meaning” (or “thoughtseeds” Kavi et al., 2024, 2025), coordinated packet complexes integrate non-contiguous groupings and thus impute meaning to indefinite stretches in the stimuli streams (unrelated bundles can have values but no meaning).

These assumptions have experimental grounding in two classes of findings: (a) movement control involves attentive rotation of population vectors in the motor cortex (Georgopoulos et al., 1986) and (b) apprehending relations involves neurons in the frontopolar cortex (Kroger et al., 2002, 2008; Kroger and Kim, 2022). Recent findings concerning organization in the behavior of neuronal populations over time (Luczak et al., 2015; Luczak, 2024) appear to provide further support to the assumption that neuronal packets serve as the core functional components of cognition (note that in Luczak et al., 2015 the term “neuronal packet” denotes streams of structured activity bursts, aligning with the notion of distinct activity patterns (units, population vectors, neural letters, neural codes) encompassing diverse populations in the cortex (Pribram, 1991; Yufik, 1998a; Li and Tsien, 2017; Lin et al., 2006; Sompolinsky, 2024). The assumptions are consistent with the foundational insights in Section 1 and have partial overlap with other viewpoints, including those summarized in Section 2. The remainder of this section discusses alignment between VaST, the Active Inference Theory (AIF) by Friston (2010), the Energy-Based Model (EBM) by LeCun et al. (2007), and the Theory of Neuronal Group Selection (TNGS) by Edelman and Tononi (2013), followed by a “wave-packet” integration proposal. Figure 1 illustrates procedures in the VaST architecture (*gnostron*) expressing meaning creation as a product of self-organization in resource-constrained neuronal pools (Yufik, 1998b).

**Figure 1:**
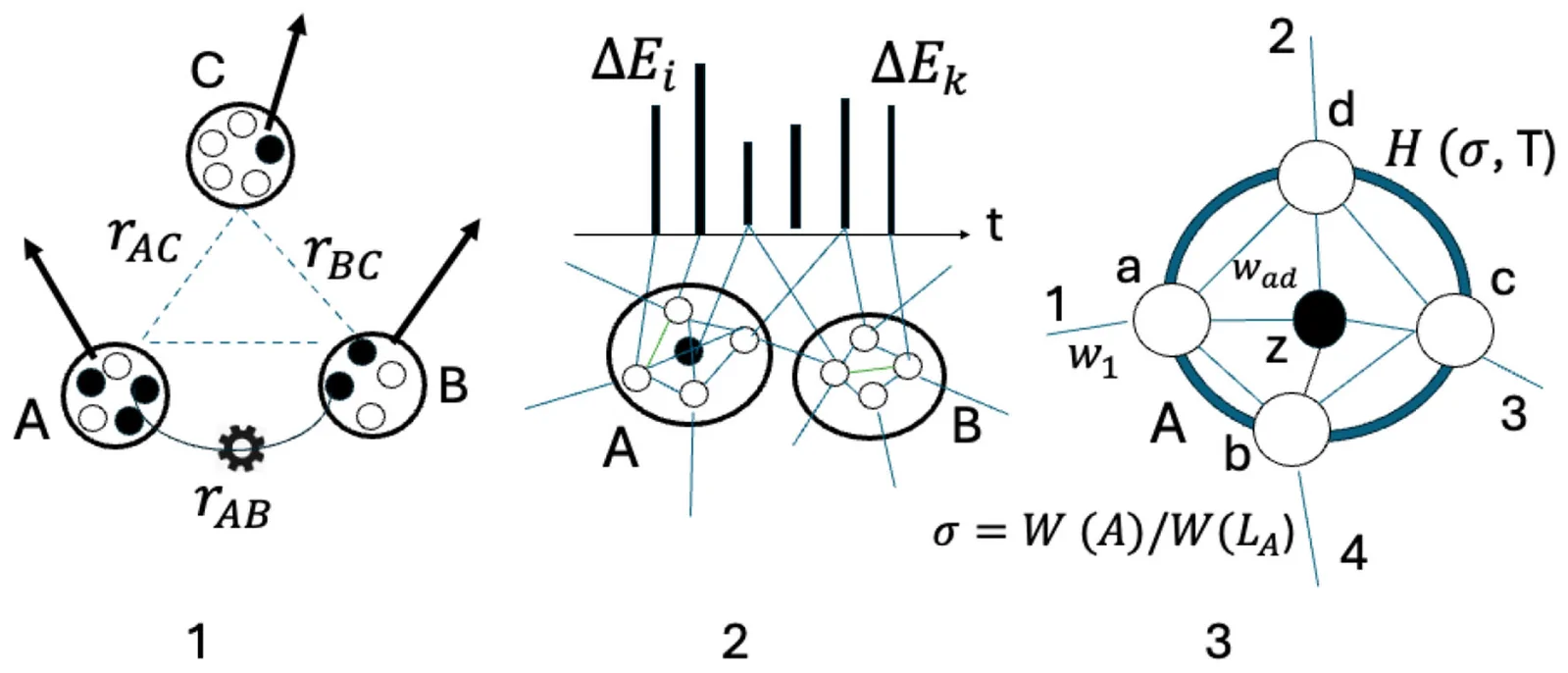
Development of virtual structures. **(1)** Hebb's triangle comprises coordinated neuronal packets, perceiving the triangle involves establishing coordination in the rotation of packets vectors accomplished with the help of relational neurons *r*_*AB*_, *r*_*Ac*_, and *r*_*BC*_. **(2)** Neurons have overlapping receptive fields responding probabilistically to different stimuli, massing neurons on stimulus *s*_*i*_ triggers energy deposit Δ*E*_*i*_, co-allocated neurons are connected by Hebbian links. Mobilizing and allocating (firing) neurons expends energy, searching for co-allocation candidates dissipates energy, the system seeks to maximize intakes while minimizing costs and losses. **(3)** Packet A consists of neurons a, b, c, d, and z connected by internal links to each other and by a link cut set (or link blanket) 1, 2, 3, and 4 to the rest of the network (removing the link blanket severs the packet from the network). Energy barrier on the node blanket (a, b, c, and d) establishes a regulated dependency: entering and exiting the packet requires energy in the amount determined by the height of the barrier H(σ, *T*), here σ is the ratio of the packet weight to that of its link blanket and T is temperature (reflects the intensity of focused (attentive) and diffused energy delivery to the packet, focused delivery is regulated by the subjective importance (value) attached to the packet).

Formation of energy barriers involves phase transitions in the network such that intersections in the link cut and node (a, b, c, and d) form a surface (as in a raindrop or snowflake) where surface tension holds the packet together and resists both entering into and exiting from the packet.

Please note that this conceptualization resonates with the “snowflake” insight in Chomsky (2014) and Berwick and Chomsky (2016) envisioning physical self-organization (as in snowflakes) at the foundation of the language capacity. The inherent compositionality of both language processes and packet manipulations suggest a substantive alignment between the viewpoints (think of allocation by packets as selecting “letters” and combining them into “words” and “phrases” matched dynamically to the stream). Also, the non-linear allocation process in Figure 1(2) appears to explain the non-linearity of text processing that has been unaccounted for in the conventional theories (Yufik and Yufik, 2026).

**Figure 2:**
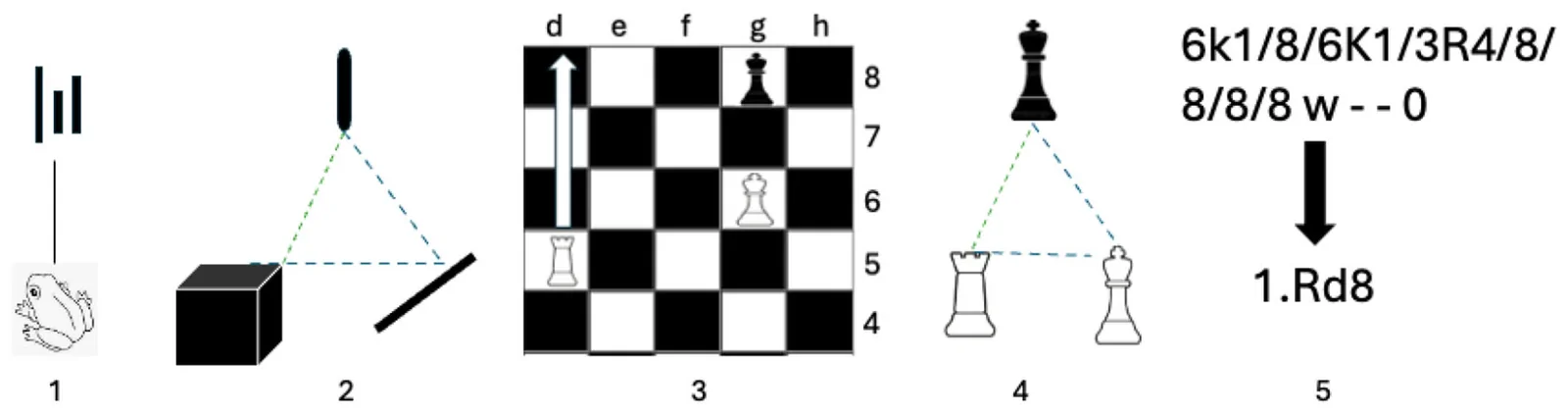
Evolution of intelligence from primitive animals to machine learning. **(1)** Primitive animals emit fixed responses to fixed bundles of contiguous stimuli (a frog shots its tongue at moving objects falling within fixed velocity, distance and size ranges). **(2)** Advanced animals (apes, some avians) possess rudimentary capacities for constructing responses involving coordinated manipulations of a few objects (a chimpanzee climbs a box and uses a stick to reach a hanging fruit). **(3)** To figure out the 1-move checkmate, one needs to visualize advancing White rook and attacking Black king simultaneously with apprehending that the White king blocks all the escape routes. **(4)** Imagining coordinated manipulation of the pieces imputes meaning to their configuration. Such imputing of meaning does not involve symbols that signify the pieces and their location on the board (as in Kroger et al., 2008). **(5)** Statistical learning accumulates records of the past games, identifies statistical regularities and applies them to compute (predict) responses to the given symbol sequences. The process is oblivious to the meaning of the sequences and to what the individual symbols stand for. On that fact, the “1. Rd8” response, although a correct one, manifests no more understanding than that of a frog shooting its tongue at a flying insect.

Perceiving triangular shapes in Figure 1(1) requires inter-packet coordination through “chance anatomical interconnections” (Hebb, 1947, p. 97). By contrast, understanding triangles involves deliberately imagining changes in the elements and experiencing coordinated responses to such changes across the construct. To emphasize: models are synergic structures (Latash, 2010, 2021) in that imagining changes in any angle automatically brings to imagination the corresponding changes in the other two angles. Awareness that the sum of the interior angles is a constant precedes formal proofs. In this way, understanding transcends proofs: the idea of the sum invariance could not come to mind without the model in place. Figure 2 will help summarizing and illustrating these notions.

The following contentions will put into sharp relief the above notions. (A) Prediction capacity has been of marginal importance in the evolution of intelligence before the emergence of Sapience. Animals respond to genetically fixed stimuli bundles [as in Figure 2(1)], predators possess fixed anticipatory and pursuit repertoires that are tuned to particular bundle compositions (types of prey) and are amenable only to narrow, feedback—driven adjustments (learning). (B) Submitting the results in Figure 2(5) to the Turing test logic (roughly, if 1. Rd8 is a good move than admit that procedures in Figure 2(5) understand chess) amounts to playing “peekaboo” with oneself, by pretending that the actual process [Figure 2(4)] or the procedure [Figure 2(5)] is unknown. If the former—consult a chess book, if the latter—read the code. A frog with a modicum of understanding would be able to imagine that a sitting insect can takeoff and fly, thus overriding the deficit that the history of exposure to such highly probable event sequences failed to correct. The point is that designing variations of Turing test to assess understanding levels achieved in machine learning can hardly lead to new revelations while diverting attention from the key problem: what are the biological mechanisms of understanding and how can they be approximated in tractable procedures. The VaST proposal identifies self-organizing models anchored in latent relational structures as a solution candidate, as argued in Yufik (2026) and the remainder of this article.

Reaching the fruit by a chimpanzee in Figure 2(2) requires manipulation of the packets until their “co-instantaneous co-ordination” (Piaget, 1978, p. 219) is reached, which goes beyond perception but does not amount to understanding (the animal would not envision that shorter sticks demand higher boxes, etc.). Solving the position in Figure 2(3) requires imagining maneuvers of the pieces concluding in the checkmate coordination. The model enabling such maneuvering is a virtual structure, that is, a superposition of the possible “a king and a rook against a king” games that guides the execution of particular games. In the structure, thinking of moving a piece is concomitant with the awareness of how that move constrains those afforded by the other pieces. Relations in the model are meaningful (e.g., *r*_*AB*_ denotes A *attacks* B), having a dual effect of allowing an indefinite multitude of admissible combinations while, at the same time, reducing that multitude to a few meaningful ones. When the model is formed, it becomes a unit in the “a king and a major piece against a king” repertoire which is a unit in the endgame repertoire, and so on (Alburt and Lawrence, 2015).

Models eliminate degrees of freedom in their constituents and become new units possessing their own identity and characteristics (e.g., “a king and a major piece” can force checkmate against a lone king while “a king and a minor piece” cannot). A crucial distinction between the “reaching fruit” and “checkmate” scenarios lies in the fact that one can find objects in the world but cannot find chess games. Accordingly, prosecution of the former scenario can be guided by physical feedback while prosecution of the latter one is guided by understanding (Yufik and Yufik, 2026).

The VaST proposal aligns closely with the Active Inference Theory (AIT) (Yufik and Friston, 2016; Ramstead et al., 2021). The graph-theoretic notions of link cuts and nodes in Figure 1 align with that of Markov blanket in expressing the intuition of a “membrane” separating a unit from its surrounds. Procedures in Figure 1 can be re-stated in terms of Bayesian inference (e.g., a rewarded allocation of neuron z suggests the presence of an object type A modeled by packet A, the probability of z is determined by the strength of its membership in A while the probability of A is a function of barrier characteristics in the terrain). Note that relational links encounter energy barriers but circumvent the blankets (think of 3D links between points inside 2D shapes).

The operation of forming relations is consistent with the idea of reentrant mappings between neuronal groups envisioned in the TNGS. Figure 1(1) integrates the ideas and suggests that (a) establishing relations engages the mechanism of reentrant mapping, (b) the mechanism operates across indefinite patches in the cortex and thus allows connecting stimuli groupings across indefinite stretches in the stream, and (c) employing the mechanism is contingent on the availability of relational neurons. To appreciate this critical limitation, imagine a reward tied to a rope with the other end left in a cage: monkeys pull in the rope while dogs fail the task (Koehler, 1951). The rope is in plain view and dogs are capable of pulling it—the solution is not independently grasped because the natural mechanism is lacking (but can be drilled in to form artificial automatisms).

The VaST framework suggests complementarity of the AIT and EBM approaches, as follows. The former seeks minimization of Variational Free Energy (VFE; roughly, a measure of discrepancy between the predicted inputs and the actually received ones) while the latter minimizes energy *E* (*X, Y*) that measures compatibility in the array comprised of input values *X* and output values *Y* [the higher the degree of compatibility, the lower the energy, *Y* = argmin_*y*∈*Y*_*E* (*y, X*)]. Insightfully, the approach recognizes *latent variables Z* that belong to neither the input nor the output: “for example, in handwriting recognition, the correct segmentation of the sentence into words and characters is never given to us, but it must be inferred in order to recognize the sentence. The recognition process can be seen as simultaneously searching for the best answer and the best segmentation” (LeCun et al., 2007), entailing energy minimization over all three variables.

$$\begin{array}{l} Y=argmin_{y\in Y}\underset{z\in Z}{\min}E\left(z,\;y,\;X\right) \end{array}$$ (1)

Return to the allocation procedures in Figure 1: the procedures maximize compatibility between the stimuli stream (*X*) and response configurations (*Y*) and employ a general segmentation mechanism comprised of self-organizing neuronal packets, self-organizing models and a hierarchy of latent relations. That is, the suggestion is that *latent variables Z* in EBM align with the *latent relational variables* *r*_*AB*_ in VaST. Think of a crossword puzzle: awareness alternates between the meaning of the words and the symbol strings that are fitted together regardless of the meaning. The EBM process is concerned with the latter aspect of the puzzle, accounting for the meaning aspect would allow advancing from recognizing sentences to understanding them and from the meaningless tapestries in crossword solutions to meaningful models. Arguably, the AIT and EBM conceptualizations complement each other in that improved compatibility required by EBM entails prediction error minimization required by the AIT, and vice versa (*min VFE* ⇆ *min E*). Both criteria are subsumed in maximizing energy inflows while minimizing costs and dissipative losses in the system. The following integration proposal supports this contention.

When monkeys are trained to touch targets, neuronal populations responsible for the stimulated surface of the trained finger expand their occupation area in the cortex and become over two times larger than the corresponding populations in other fingers. Significantly, such expansions occur in monkeys actively engaged in (paying attention to) the task while there were no such expansions in monkeys touched in the same finger surface while attending to other stimuli (Polley et al., 2006; Xerri et al., 1999). The point is that accumulation of experiences under varying conditions in diverse tasks produces dynamically expanding and contracting packets in continuously re-adjusting cortical networks—how such re-adjustments get reconciled throughout the cortex? We hypothesize that the propagation of wave activities has the effect of “shaking the packets together,” causing re-distribution of neurons and the corresponding adjustments in the energy landscape. Energy delivered by the waves (proportional to the square of the amplitude) powers variations in temperature *T* and oscillations of energy barriers *H* (σ, *T*), *entailing* release of neurons in some packets and their absorption into other packets.

Think of the Brazilian nuts effect: shaking the container transports the larger pieces to the top, with the smaller ones filling the gaps and accumulating at the bottom. The organization settles when reaching the densest packing having the center of gravity at the lowest possible level. Arguably, “shaking together” packets in packet networks drives the system toward re-distributions yielding Ockham optimality in the packet sizes and contents [as small as possible but not smaller (Yufik and Sheridan, 2002)]. Neuronal avalanches emerging through self-organized criticality present another form of “shaking” (Plenz and Thiagarajan, 2007). The phase transition model with intermittent propagations across the cortex promotes the “shaking” effect (Freeman et al., 2009; Kozma et al., 2005) in alignment with the model of anticipatory synergy adjustments in motor control (Latash, 2010). Notably, the Latin *cogitare (to think)* literally means “to shake together” (Koestler, 1967). In general, “shaking” appears to deliver consolidation in the packet system and its accommodation to the products of assimilation [i.e., maximizing the (*X, Y*) compatibility].

To summarize: we hypothesized that progress in AI will involve transition to machine understanding, discussed the state-of-the-art to substantiate the hypothesis and argued that future advances are predicated on apprehending the principles allowing the human system to deliver the unparalleled richness and power of the understanding capacity while operating on miniscule energy budgets. In conclusion, we repeat the invitation to review the trilogy and continue the discussion in the second volume, suggesting the following conversation starters:

- The Libet phenomenon (delays between the onset of neuronal activities initiating finger movements and the awareness of such initiation) casts no doubts on the existences of free will: bringing into awareness packet contents requires overcoming energy barriers. In a continuous volitional act, the content-agnostic experience of entering and exiting packets is different from the reportable, content-sensitive experience of rotating packet vectors.
- The engine of intelligence is a modality-agnostic energy optimization mechanism (Figure 1) manifested in the compositional and non-linear operations on streams in different modalities [scene understanding, text understanding, music understanding, situation understanding, problem solving in “streams of thought” (James, 1890, p. 223)].
- Integration of symbolic and subsymbolic modalities poses no binding problem in packet networks. Making choices necessitates navigation of energy terrains, symbol-holding packets make the navigation energetically feasible by providing cheap over-passes for the content-holding packets in the energy valleys. Language facilitates “streams of thought,” as needed for forming relations. Learning in the perceptron involves no such activities and thus has neither a need nor room for symbol manipulation. Hence, the problem.
- McCullough-Pitts (MP) digital neurons are different from Hebb (H) neurons: MP neurons compute and telegraph messages while H neurons convey energy and matter and self-organize into functional wholes. In machine understanding, MP neurons are programmed to approximate processes in H neurons responsible for the understanding capacity.
- The idea of “co-instantaneous co-ordination” aligns with that of quantum entanglement. On that view, both understanding and consciousness “would be some manifestation of the quantum-entangled internal cytoskeletal state and of its involvement in the interplay (Objective Reduction) between quantum and classical levels of activity” (Penrose, 1994; p. 376). The architecture of intelligence includes microtubules (quantum level), neurons (microlevel), packets (mesolevel), and models (macrolevel). Collapse at the quantum level is facilitated by wave activities at the mesolevel and manifests in grasp at the macrolevel.
- The Information Integration Theory (IIT) (Tononi, 2008) uses a measure of functional clustering in the neuronal pool (cluster index) to differentiate between normal and pathological states, for example, schizophrenia (Edelman and Tononi, 2013). Correspondence between the VaST packet groupings and IIT cluster index variations would indicate that pathological deviations can be caused by biophysical abnormalities in the neurons and/or system-level impairments in the regulation of energy landscapes (e.g., schizophrenic fragmentation).
- Understanding guides internal processes decoupled from the sensory-motor feedback. Machine understanding requires self-cognizance sufficient for assessing own degree of understanding: “I understand that” or “This data makes no sense.” Self-cognizant agents can explain themselves (as opposed to AI procedures explaining other AI procedures, as in Explainable AI) and are inherently understandable to humans (Yufik and Yufik, 2026).
- Shared understanding dynamically shapes contents and cuts down the amount of communication between machine agents and in the human-machine teams.
- Hardware for machine understanding will be based on self-organizing memory and will allow emulation, within limits, in moderate-capacity conventional platforms.

Finally, a new meaning of the old parable: a true image of the elephant comes not from the descriptions of body parts but from imagining how such parts coordinate in the moving animal.
